# Cost-effectiveness evaluation of glucosamine for osteoarthritis based on simulation of individual patient data obtained from aggregated data in published studies

**DOI:** 10.1007/s40520-019-01138-1

**Published:** 2019-02-12

**Authors:** Olivier Bruyère, Jean-Yves Reginster, Germain Honvo, Johann Detilleux

**Affiliations:** 1 Department of Public Health, Epidemiology and Health Economics, WHO Collaborating Centre for Public Health Aspects of Musculo-Skeletal Health and Ageing, University of Liège, 4000 Liège, Belgium; 2 Prince Mutaib Chair for Biomarkers of Osteoporosis, Biochemistry Department, College of Science, King Saud University, Riyadh, 11451 Saudi Arabia; 3 Department of Veterinary Management of Animal Resources, University of Liège, Liège, Belgium

**Keywords:** Osteoarthritis, Cost-effectiveness, Glucosamine

## Abstract

**Background:**

The economic evaluation of treatments usually requires access to individual patient data, which is difficult to obtain. Moreover, in osteoarthritis, health utility scores are unavailable and can be assessed only using a validated equation model based on various clinical data. We aimed to develop and validate a methodology to simulate individual health utility scores from aggregated clinical data available in published studies to calculate the cost-effectiveness of different glucosamine preparations (i.e., crystalline glucosamine sulfate, glucosamine sulfate, and glucosamine hydrochloride) used for osteoarthritis.

**Methods:**

We developed a method to simulate individual utility values and validated the model by comparing the results obtained with the simulation and the results of one trial where the utility scores are available. Then, we simulated the utility scores of 10 published trials that used different glucosamine preparations. The utility estimates were used to calculate the quality-adjusted life year (QALY) using the area-under-the-curve method. Costs were for the glucosamine product only. The incremental cost/effectiveness ratio (ICER) was then calculated.

**Results:**

The values of utility scores calculated from data sources and those simulated with the model were similar. From 10 studies where utility was simulated, four used crystalline glucosamine sulfate, and six used other formulations. The ICER revealed that compared to placebo, crystalline glucosamine sulfate only was cost-effective at all time points and up to 3 years with a median ICER of 5347.2 €/QALY at month 3, 4807.2 €/QALY at month 6 and 11535.5 €/QALY at year 3. The use of other formulations was not cost-effective.

**Conclusion:**

Using a new model to simulate individual health utility scores of patients included in ten published trials, ICER analysis showed that the use of crystalline glucosamine sulfate is cost-effective, while other formulations were not. The results confirm the importance of the formulation of glucosamine products.

## Introduction

Osteoarthritis (OA) is a major public health problem because of its current and future prevalence, its impact on mortality and morbidity and the associated healthcare cost [1]. Pharmacological and non-pharmacological treatments are currently available and their effects in reducing the symptoms of OA or increasing the quality of life of OA patients have been widely assessed by different scientific organisations [2–4]. A treatment algorithm for the management OA was also proposed, which provides practical guidance for the prioritisation of interventions [5]. In this algorithm, the use of symptomatic slow-acting drugs for osteoarthritis (SYSADOA) as the first pharmacological therapy is recommended. However, some international scientific societies do not recommend the use of SYSADOAS in their most recent guidelines [2, 4, 6]. Indeed, there are many different agents in the class of SYSADOAs, including glucosamine, chondroitin, diacerein, and avocado soybean unsaponifiables, and not all are supported with a high level of clinical efficacy data, nor supported by the same degree of recommendation in clinical guidelines. Although more studies are needed to further substantiate their precise effects, some evidence is available with the use of glucosamine and chondroitin as SYSADOAs with an impact on both symptoms and structure in the long term. Multiple formulations of glucosamine and chondroitin are available as both prescription-grade products and nutritional supplements, and these differences have been hypothesised as being an important driver of the discrepancy in the level of recommendation of SYSADOAS between guidelines [7]. Indeed, although all preparations may claim to have a therapeutic effect, not all are supported by clinical evidence [8]. For example, with glucosamine, an independent meta-analysis has shown that in trials using a specific formulation of glucosamine, the prescription crystalline glucosamine sulfate (pCGS) had a better outcome on pain than did other preparations of glucosamine [9].

In a world with limited resources and health care budgets, it is important to allocate scarce resources efficiently and consequently, to develop effective treatments and efficient strategies. Economic evaluation is a method for comparing different strategies in terms of cost (e.g., intervention costs and disease costs) and consequences [e.g., life years or quality-adjusted life year (QALY)]. These evaluations play a growing role in pricing and reimbursement decisions as regulatory agencies rely more and more on pharmacoeconomic data to make decisions about limited resources [10]. To the best of our knowledge, only one clinical trial has explored the cost-effectiveness of glucosamine (and in this particular case the pCGS formulation) compared with that of paracetamol and placebo in the treatment of knee OA [11]. The authors concluded that compared with paracetamol and placebo, pCGS was a highly cost-effective therapy to treat patients diagnosed with knee OA. However, another research group using a cohort simulation model based on clinical data available from five systematic reviews and one clinical guideline showed that there was evidence that glucosamine sulfate shows some clinical effectiveness in the treatment of OA of the knee [12]. However, in this economic evaluation the authors highlighted the need for further research since they showed that estimates were imprecise and subject to a degree of decision uncertainty.

To further advance the economic evaluation of glucosamine, we should theoretically have access to individual patient data. Unfortunately, most of the time, access to these data is not possible because of technical, legal or patient willingness issues. Consequently, we decided to develop and validate a method to simulate individual health utility scores based on the aggregated clinical data provided in published papers of clinical trials. Based on this method of simulation, we performed some cost-effectiveness analyses based on trials that used different formulations of glucosamine.

## Materials and methods

### Development of the simulation model

To simulate individual values, we had to solve two major issues. The first issue is that health utility scores are not directly assessed in most OA studies in contrast to WOMAC (i.e., an OA specific health-related quality of life questionnaire) scores. Interestingly, Grootendorst et al. [13] have developed a linear regression model to estimate utility scores based on the age of the patient, the number of years since he/she was diagnosed with OA and the three different WOMAC subscales scores. The second issue is that only summary statistics (mean, median, standard deviation, covariance or confidence interval) of age, number of years since OA and WOMAC scores are reported in most OA trials.

To overcome these issues, we propose the following procedure to obtain individual health utility scores:

- Use the SIMNORMAL procedure of SAS and published summary statistics to simulate individual values for the WOMAC indexes, age and years since OA. The SIMNORMAL procedure performs conditional and unconditional simulation for a set of correlated normal or Gaussian random variables.
- Discard simulated values outside the following permissible ranges: 0–20 for pain, 0–68 for function, 0–8 for stiffness, 0–100 for age and 0–100 for years since OA diagnosis.
- Compute individual female utility scores from the equation provided in the paper by Grootendorst (considering that the parameter “female” in the equation of Grootendorst takes the value of 1 if the patient is a woman).

To validate the procedure, we tested it on data from the only study on glucosamine in which individual health utility values were published [11] and for which we have access to the individual values for WOMAC scores, age and years since OA diagnosis, at baseline and after 3 months of treatment. We computed means and standard deviations for and between these values with the CORR procedure of SAS at baseline, after 3 months and for subjects in the placebo and pCGS groups. Next, we compared simulated and published values.

### Simulation of individual health utility scores

We used the procedure to simulate individual health utility scores for all clinical trials cited in the meta-analysis of Eriksen et al. [9] that have used the WOMAC. Consequently, we simulated individual health utility scores from ten trials (4 using pCGS and 6 using other forms of glucosamine) [14–23]. When available, means and SD were extracted from published articles, after correction for the scales (to be on the scale for WOMAC indexes as the one used in the equation of Grootendorst). We replaced missing data in the summary statistic of published studies (i.e., sometimes data, such as the standard deviation or a specific variable such as years since OA, were unavailable) with data from the study used to develop and validate the procedure [11]. We simulated a total of 20,000 patients in each study (10,000 glucosamine and 10,000 placebo), and data were examined by two independent experts.

### QALY assessment

The utility estimates were used to calculate the QALY using the area-under-the-curve method [24] that is the weighted average of time spent in the study and utility value. If more than one study was available for a particular time (e.g., 3 months), we weighted each study according to the number of subjects included in the trial.

### Costs of glucosamine

Since there are major differences in products, reimbursement strategies, daily dosage, among other factors, the range of costs of glucosamine products varies widely. To get an official overview, we could access the official IMS Health data, updated at December 2017, related to the main selling prices of the different formulations, in the different countries. Since the data is reported in both local currency and US dollars, it was possible to report data in US dollars and then in Euros with homogeneous currency exchange rate. Obviously, we separated pCGS and other forms of glucosamine. Since the price range was quite wide, to reduce variability and define a reasonably weighted average price for each of the two forms of glucosamine, the following methodology was devised: after calculation of the overall average price, all prices that were > 50% lower than this average price were excluded, and the new average of the remaining prices was calculated and defined as the “higher” value of the cost-range. In the same way, we started again with all prices and excluded those that were > 50% higher than the overall average, and calculated a new average of the remaining prices, which was defined as the “lower” value of the price range. Consequently, we achieved a median cost of 0.79 €/day for pCGS (with low and high prices of 0.65 €/day and 0.88 €/day, respectively) and a median cost of 0.55 €/day for other forms of glucosamine (with low and high prices of 0.45 €/day and 0.66 €/day, respectively). The price of placebo was considered 0 €.

### Cost-effectiveness analysis

The incremental cost/effectiveness ratio (ICER)—a measure of the additional cost per unit of health gain—was then calculated. The underlying calculation for the ICER comparing glucosamine products vs. placebo in patients with knee OA was equal to (average cost glucosamine − average cost placebo)/(average effect glucosamine − average effect placebo) where costs were measured in Euros, and effects were measured in QALY.

### Sensitivity analysis

Because of the different time points used in different studies (i.e., from 2 months to 3 years), we decided to use data from longer studies at all time points. For example, for a 3-year study, we considered an 8.3% of the global effect at month 3 (3/36 × 100 = 8.3%) and a 16.7% of the global effect at month 6 (6/36 × 100 = 16.7). Obviously, we acknowledge that this approach could reduce the potential (cost)-effectiveness of the treatment since it has been shown that at least 3 months is needed to begin seeing an effect with most of the SYSADOAs.

## Results

From the study of Herrero-Beaumont et al. [16], the simulated mean health utility score was 0.598 at baseline and 0.676 at the end of the study in the glucosamine group. These values were respectively 0.602 and 0.651 in the placebo group. The change over time was 0.08 in the glucosamine group and 0.05 in the placebo group. Interestingly, these values are completely similar to the previously published results (i.e., 0.59 and 0.60 at baseline and a 0.08 and 0.05 change over time in the glucosamine group and the placebo group, respectively) suggesting that the simulation model provides reliable utility value [11].

The differences between the glucosamine and placebo groups in the changes over time of the simulated utility scores of all clinical trials that have used the WOMAC are presented in Table 1. As expected from previously published WOMAC results [9], the differences in changes in utility values were more often in favour of glucosamine in trials that used pCGS than in those that used other formulations.

**Table 1:** Health utility changes in the studies using pCGS or other forms of glucosamine

	Type of glucosamine	*N*	Duration (month)	Health utility changes in the glucosamine group	Health utility changes in the Placebo group
Giordano et al. [17]	pCGS	60	3	0.135	− 0.0742
Beaumont et al. [16]	pCGS	210	6	0.0785	0.0315
Reginster et al. [14]	pCGS	212	36	0.1606	0.1427
Pavelka et al. [15]	pCGS	202	36	0.0487	0.0207
Houpt et al. [18]	Other form of glucosamine	101	2	0.0293	0.0129
McAlindon et al. [19]	Other form of glucosamine	205	3	− 0.0465	− 0.0339
Frestedt et al. [22]	Other form of glucosamine	35	3	0.1629	0.0613
Chopra et al. [25]	Other form of glucosamine	70	3	0.0016	0.1232
Cibere et al. [20]	Other form of glucosamine	137	6	0.0096	0.0006
Clegg et al. [21]	Other form of glucosamine	630	6	0.0242	0.0383

**Fig. 1:**
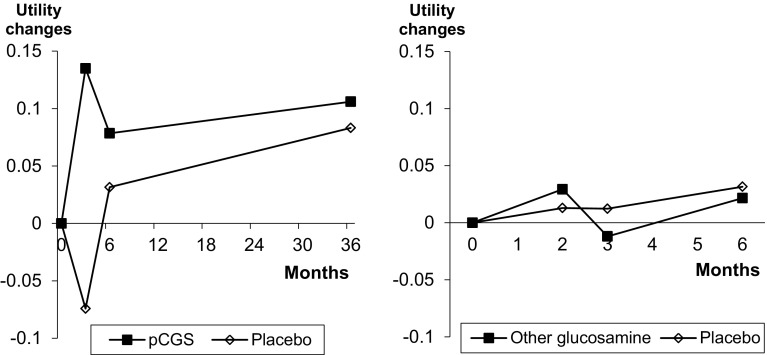
Health utility evolution in trials having used pCGS or other forms of glucosamine

The evolution of the health utility scores among studies that used pCGS or other forms of glucosamine is presented in Fig. 1. The utility score always improved when pCGS was used while the results are much more variable when other formulations of glucosamine were used.

The calculation of the ICER among studies having used pCGS show that the use of this formation is highly cost-effective compared to placebo, regardless of the price of the treatment (Table 2). The ICER calculation also shows that pCGS is already cost-effective after 3 months of treatment and up to 3 years after the initiation of the treatment.

**Table 2:** Incremental cost-effectiveness ratio results of studies having used pCGS

	At 3 months	At 6 months	At 36 months
QALY change pCGS	0.016875	0.0435625	0.27418931
QALY change placebo	− 0.009275	− 0.0146125	0.12872929
Median cost pCGS	139.83	279.66	1677.96
Median ICER	5347.2	4807.2	11535.5
Lowest cost pCGS	115.05	230.1	1380.6
Lowest ICER	4399.61759	3955.30726	9491.2675
Highest cost pCGS	155.76	311.52	1869.12
Highest ICER	5956.40535	5354.87752	12849.716

When looking at other forms of glucosamine, notably, if the ICER results could be considered cost-effective at 2 months (based on one single study), the results no longer show the cost-effectiveness of these other formulations of glucosamine at 3 months (Table 3). Moreover, at 6 months of treatment, from a health economics perspective, placebo is even better than these formulations. None of these results are substantially influenced by the costs of glucosamine.

**Table 3:** Incremental cost-effectiveness ratio results of studies having used other forms of glucosamine

	At 2 months	At 3 months	At 6 months
QALY change other glucosamine	0.002344	0.00303613	0.00423555
QALY change placebo	0.001032	0.0020409	0.00752699
Median cost other glucosamine	33	49.5	99
Median ICER	25,152.4	49,737.4	Placebo better
Lowest cost other glucosamine	27	40.5	81
Lowest ICER	20,579.2	40,694.2	Placebo better
Higesth cost other glucosamine	39.6	59.4	118.8
Highest ICER	30,182.9	59,684.9	Placebo better

**Fig. 2:**
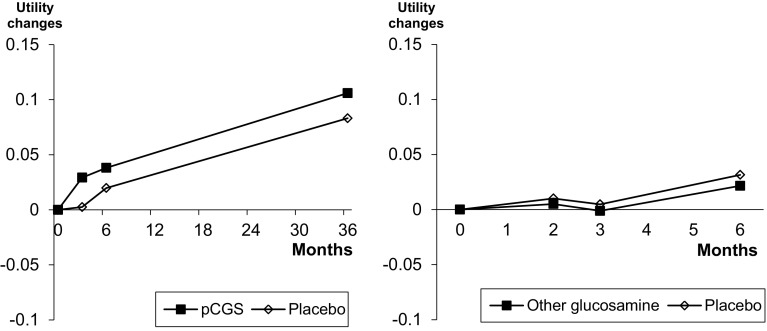
Health utility score evolution using data from longer studies at all time points among studies that used pCGS or other forms of glucosamine

The results of sensitivity analyses, using data from longer studies at all time points, are presented in Fig. 2 and Tables 4 and 5. For pCGS, the health utility change is always in favour of the treatment compared to placebo and the ICER shows a cost-effectiveness that increases over time. Using other formulations of glucosamine, no meaningful difference in utility scores is observed compared to placebo and the cost-effectiveness analyses confirm the absence of economic interest of these formations at all time points.

**Table 4:** Incremental cost-effectiveness ratio results of studies that used pCGS–sensitivity analyses

	At 3 months	At 6 months	At 36 months
QALY change pCGS	0.00365489	0.01207722	0.19225352
QALY change placebo	0.00031524	0.00310523	0.13181955
Median cost pCGS	139.83	279.66	1677.96
Median ICER	41,869.6	31,170.3	27,765.1
Lowest cost pCGS	115.05	230.1	1380.6
Lowest ICER	34,449.7143	25,646.468	22,844.7678
Higesth cost pCGS	155.76	311.52	1869.12
Highest ICER	46,639.6132	34,721.3721	30,928.301

**Table 5:** Incremental cost-effectiveness ratio results of studies that used other forms of glucosamine–sensitivity analyses

	At 2 months	At 3 months	At 6 months
QALY change other glucosamine	0.0004075	0.00056492	0.00311919
QALY change placebo	0.00080951	0.00140589	0.00595049
Median cost other glucosamine	33	59.4	118.8
Median ICER	Placebo better	Placebo better	Placebo better
Lowest cost other glucosamine	27	40.5	81
Lowest ICER	Placebo better	Placebo better	Placebo better
Highest cost other glucosamine	39.6	59.4	118.8
Highest ICER	Placebo better	Placebo better	Placebo better

## Discussion

In this study, we developed a model that could simulate individual patient data based on aggregated data found in published scientific papers. Indeed, most of the economic analyses require an access to individual patient data, which is unfortunately very difficult. Our model simulated all data needed to calculate the utility score (i.e., age, years since OA diagnosis, and WOMAC subscales). Using one validation study, our simulation model was able to provide summarised data similar to what could be found in the literature in term of utility score. It is also interesting to note that the utility score calculated with the simulation model is within the range observed in patients with osteoarthritis [26].

With the individual patient data simulated from ten randomised controlled trials, we were able to calculate the ICER of different formulations of glucosamine. Our results showed that the use of pCGS only was cost-effective compared to placebo. To the best of our knowledge, only one clinical trial has been conducted, to assess, in post hoc analysis, the cost-effectiveness of glucosamine. Using data form the GUIDE study, Scholtissen et al. showed that the use of pCGS was cost-effective compared to placebo but also to paracetamol [11]. However, one other study has assessed the health care utilisation following the use of pCGS [27]. In that particular 8-year observational study following a 3-year randomised controlled trial, the authors showed that patients formerly on pCGS had recurred with less symptomatic medications and the use of other health resources than did those from the placebo group during the last year of follow-up.

In this study, we also confirm the difference of (cost-)effectiveness among different glucosamine preparation. Indeed, our results highlight that pCGS, but not the other formulations of glucosamine is cost-effective. These results are supported by the recent meta-analysis of Eriksen et al. showing that the reduction of pain and the improvement in function was observed only with pCGS and not with the other types of glucosamine [9]. Glucosamine exists in different forms. However, all these products have not been characterised in the same way in terms of quality, pharmacokinetics and equivalence of human biological fluid levels with mechanistic data [28].

We have to acknowledge major limitations in our study. First, our simulation model has been validated using data from a single study only. Second, we did not have access to raw data to perform the ICER evaluation. Third, the cost evaluation was limited to the cost of the treatment and other costs have to be included to achieve a full health economics evaluation (e.g., the health care resources, the patient and family resources, the productivity costs and the others sectors resources). Fourth, some data needed to calculate the utility were unavailable in some publications, and we had to replace these data by data from the trial used to validate the model. Fifth, we had no direct assessment of the utility score and had to use the regression model developed and validated by Grootendorst et al. to obtain an estimation of the utility based on the WOMAC data [13], which is only one of the tools to assess quality of life in musculoskeletal health [29].

In conclusion, we confirm the superiority of pCGS over the other glucosamine formulations in terms of efficacy and cost-effectiveness. These results should be confirmed in other studies taking into account compliance to therapy, the other costs of OA management and a direct assessment of utility.
